# Antioxidant Potential, Antinutrients, Mineral Composition and FTIR Spectra of Legumes Fermented with *Rhizopus oligosporus*

**DOI:** 10.17113/ftb.59.04.21.7319

**Published:** 2021-12

**Authors:** Barinderjeet Singh Toor, Amarjeet Kaur, Param Pal Sahota, Jaspreet Kaur

**Affiliations:** Punjab Agricultural University, Ferozepur Road, Ludhiana, Punjab-141004, India

**Keywords:** *Rhizopus oligosporus*, fermented legumes, antioxidant potential, nutritional and antinutritional profile

## Abstract

**Research background:**

Legumes are superior sources of macro- and micronutrients which can be further enhanced by fermentation. This can assist in addressing the food security concerns. The present study aims to determine the effect of fermentation by *Rhizopus oligosporus* on nutritional and antinutritional composition of some commonly consumed legumes.

**Experimental approach:**

Chickpea (kabuli and desi), pigeon pea and soybean were fermented with *Rhizopus oligosporus* (at 34 °C for 52 h), dried at 45 °C for 16-18 h and milled. Antioxidant potential, phenolic composition, antinutrients, mineral composition and FTIR spectra of fermented and unfermented flour samples were evaluated.

**Results and conclusions:**

Fermentation significantly (p<0.05) enhanced the total phenolic and flavonoid contents, and antioxidant properties (radical scavenging activity, reducing power, ferric reducing antioxidant power and metal chelation) of kabuli and desi chickpeas, and soybean. Although fermented pigeon pea exhibited excellent antioxidant properties, the effect of fermentation on such properties was either minimal or insignificant. Additionally, quantification of specific phenolics using HPLC showed higher mass fractions of certain compounds such as chlorogenic, *p*-hydroxybenzoic, gallic and vanillic acids in fermented legumes. Mass fraction of phytic acid in all the fermented legumes was reduced (p<0.05), while trypsin inhibition increased (p<0.05). In kabuli and desi chickpeas, and pigeon pea, saponin mass fraction increased (p<0.05) while it decreased in soybean. Tannin mass fraction increased (p<0.05) in desi chickpea, pigeon pea and soybean and decreased (p<0.05) in kabuli chickpea. Furthermore, fermentation enhanced the content and estimated bioavailability of minerals. FTIR spectrum of fermented and unfermented legumes showed the presence of several functional groups and modifications in the molecular structure after fermentation.

**Novelty and scientific contribution:**

To our knowledge, this is the first study where legume (kabuli and desi chickpeas, pigeon pea and soybean) fermentation by *Rhizopus oligosporus* has been assessed for nutritional and antinutritional profile and FTIR spectra. We concluded that the treatment resulted in an optimal balance of nutrients and antinutrients. The process proved to be a potential tool for tackling the concerns of nutritional security, and thus can be proposed for the development of novel legume-based functional foods.

## INTRODUCTION

Growing population combined with climatic changes poses a huge threat to food and nutrition securities. In such a scenario, plant foods, particularly legumes are playing an important role ascribed to their rich nutritional composition that includes quality proteins, essential minerals and amino acids as well as carbohydrates (*1*, *2*). Several epidemiological studies have concluded that the regular consumption of legumes prevents the body from various health conditions like coronary heart disease, diabetes and colon cancer (*3*). Such beneficial effects are mediated by the presence of phenolics, bioactive peptides, amino acids and vitamins (*4*). However, legumes contain certain antinutrients including phytic acid, trypsin inhibitor, saponins and tannins, which reduce the bioavailability of nutrients through complexation (*5*).

Several innovative strategies have been developed to mitigate the negative effects of antinutrients and to enhance the functionality of legumes. Fermentation is a potential technique that has been found to be effective against antinutritional factors; it also contributes to the organoleptic properties and shelf life extension (*5*). Enzymes (protease, tannase and amylase) secreted during fermentation hydrolyze complex molecular structures to simpler forms, which results in enhanced digestibility and bioavailability of nutrients including minerals (calcium and phosphorus) and limiting amino acids. Moreover, fermentation has the ability to mobilize the bound phenolics to improve the overall phenolic content and related antioxidant properties (*3*, *6*).

*Rhizopus oligosporus,* a filamentous fungus utilized in the present study, belongs to the family *Mucoraceae* and is generally recognized as safe (GRAS). *R. oligosporus* has been demonstrated as detoxifying agent against food toxins, such as ochratoxin A. Moreover, *R. oligosporus* resists the proliferation of pathogens, for instance, *Staphylococcus aureus,* through the formation of antibiotics (*7*, *8*). *R. oligosporus* has been utilized for a long time for the fermentation of soybean to produce tempeh, a traditional Indonesian soy product. Recently, substrates like chickpeas, mung beans and kidney beans have been utilized for the preparation of tempeh (*9*, *10*). The aim of this study is to elucidate the effect of fermentation on the antioxidants, antinutrients, mineral composition as well as FTIR spectra of chickpea (kabuli and desi), pigeon pea and soybean.

## MATERIALS AND METHODS

### Materials

Chickpea kabuli (genotype L 552), chickpea desi (genotype PBG 7), pigeon pea (variety AL 882) and soybean (variety SL 958) were procured from Punjab Agricultural University, Ludhiana, India. *Rhizopus oligosporus* MTCC 556 was obtained from Microbial Type Culture Collection and Gene Bank (MTCC), CSIR-Institute of Microbial Technology, Chandigarh, India. Methanol, Folin-Ciocalteu reagent, sodium carbonate, aluminium chloride, sodium hydroxide, quercetin, 2,2-diphenyl-1-picrylhydrazyl (DPPH), 2,2′-azino-bis(3-ethylbenzothiazoline-6-sulfonic acid) (ABTS), potassium persulfate, potassium hexacyanoferrate(III), phosphate buffer, trichloroacetic acid, iron(III) chloride, l-ascorbic acid, TPTZ (2,4,6-Tris(2-pyridyl)-*s*-triazine), iron(II) sulfate heptahydrate, ferrozine, acetonitrile, acetic acid, cinnamic acid, ferulic acid, chlorogenic acid, *p*-hydroxybenzoic acid, vanillic acid, gallic acid, tannic acid, ammonium iron(III) sulfate dodecahydrate, thioglycolic acid, sodium phytate, ethyl acetate, anisaldehyde, saponin, sodium chloride, bovine pancreatic trypsin, *N*-benzoyl-dl-arginine-*p*-nitroanilide (BApNA), calcium chloride and dimethyl sulfoxide (DMSO) were purchased from SRL Chemicals, Mumbai, MH, India. Trolox, syringic acid, caffeic acid and Folin-Denis reagent were procured from Otto Chemicals, Mumbai, India. Hydrochloric acid, concentrated sulphuric acid, nitric acid, perchloric acid and sodium nitrite were purchased from Molychem, Mumbai, India. The 2,2’-bipyridine and ethanol were purchased from HiMedia Laboratories Pvt. Ltd., Mumbai, India. Tris-HCl buffer and acetate buffer were purchased from Sigma-Aldrich, Merck, Mumbai, India.

### Fermentation

For fermentation, seeds were soaked overnight in water at 25 °C followed by cooking for 30 min; all except soybean, which was cooked for 1 h. Cooked seeds were inoculated with *R. oligosporus* suspension (10^9^ spore/kg) and incubated for 52 h at (34±1) °C. The cooking time and fermentation process were optimized by preliminary trials. Fermented seeds were dried in a tray dryer (model Td-12; Narang Scientific Works, New Delhi, DL, India) at (45±1) °C for 16–18 h and milled to flour (60 BSS mesh, *i.e.* 0.25 mm) using domestic mill (Saffron Home Appliances, Ahmedabad, GJ, India). To obtain unfermented flour, legumes were soaked, cooked, dried and milled similarly to fermented flour. Fermented and unfermented legume samples were kept at 4 °C in airtight pouches until analysis (*11*).

### Antioxidant properties

#### Extract preparation

One gram of each sample was refluxed twice with 20 mL of aqueous methanol (80% methanol acidified with 0.1% HCl) for 2 h. The extracts were pooled and centrifuged at 1600×*g* for 10 min (model T-8BL; Laby Instruments Industry, Ambala, HR, India). The final volume was made up to 50 mL with aqueous methanol (*γ*(sample)=20 mg/mL). All the extracts were stored in amber glass bottles at 4 °C until analysis.

#### Total phenolic content

The method reported by Singleton *et al*. (*12*) was utilized for the determination of total phenolic content (TPC). A volume of 0.5 mL of extract was taken in test tubes and diluted to 1 mL with aqueous methanol. Then, 5 mL of freshly prepared Folin-Ciocalteu reagent (10% *V*/*V*) were added, followed by the addition of 4 mL saturated Na_2_CO_3_ solution. After incubation for 2 h, the absorbance was measured at 765 nm (model Spectro-4; Laby Instruments Industry) against reagent blank. Results were expressed in milligrams of gallic acid equivalents (GAE) per gram of dry sample.

#### Total flavonoid content

Total flavonoid content (TFC) was measured according to the method given by Dini *et al*. (*13*) with certain modifications. A volume of 0.5 mL of extract and 2 mL of distilled water were mixed in test tubes followed by the addition of 0.15 mL of 5% NaNO_2_ aqueous solution. Tubes were incubated for 5 min and then 0.15 mL of 10% AlCl_3_ aqueous solution was added. After 6 min, 1 mL of 1 M NaOH was added into the reaction mixture followed by the immediate addition of 1.2 mL of water. Final reaction mixture was thoroughly mixed, and absorbance was measured at 510 nm (model Spectro-4; Laby Instruments Industry) against reagent blank. Quercetin was used as standard to express the results in milligrams of quercetin equivalents (QE) per gram of dry sample.

#### Determination of radical scavenging activities (DPPH^•^ and ABTS^+•^)

DPPH^•^ (2,2-diphenyl-1-picrylhydrazyl radical) scavenging activity was evaluated using the procedure given by Brand-Williams *et al*. (*14*) with some modifications. In test tubes, 0.1 mL of extract was mixed with 3.9 mL of freshly prepared DPPH solution (0.2 mM) followed by incubation for 30 min in the dark. Absorbance was measured at 515 nm using spectrophotometer model Spectro-4 (Laby Instruments Industry). Absolute methanol and DPPH solution were used as blank and control respectively. The standard curve of Trolox was plotted as a function of the percentage of DPPH radical scavenging activity. Results were expressed in micromoles of Trolox equivalents (TE) per gram of dry sample.

The method reported by Yilmaz-Ersan *et al*. (*15*) was used for the determination of ABTS^+•^ (2,2′-azino-bis(3-ethylbenzothiazoline-6-sulfonic acid) radical cation) scavenging activity. The 20 mM ABTS and 2.45 mM K_2_S_2_O_8_ solutions were mixed in the ratio 1:1 (*V*/*V*) and kept in the dark for 12–16 h at ambient conditions to produce stock solution. The working solution was prepared by diluting this stock solution with ethanol to an absorbance of 0.70±0.02 at 734 nm (model Spectro-4; Laby Instruments Industry). For analysis, 0.1 mL extract, 3.9 mL ethanol and 1 mL freshly prepared working solution were mixed. After 6 min, absorbance was recorded at 734 nm against ethanol. The standard curve of Trolox was plotted as a function of the percentage of antioxidant activity. Results were expressed in micromoles of TE per gram of dry sample.

#### Reducing power analysis

Reducing power was measured according to the procedure reported by Oyaizu (*16*). A volume of 1 mL of the methanolic extract, 2.5 mL of 1% potassium hexacyanoferrate(III) and 2.5 mL of phosphate buffer (0.2 M, pH=6.6) were mixed thoroughly and incubated for 30 min at 50 °C. Then, 2.5 mL of 10% trichloroacetic acid were added into reaction mixture followed by centrifugation at 1600×*g* for 10 min (model T-8BL; Laby Instruments Industry). A volume of 2.5 mL of supernatant was diluted with 2.5 mL distilled water and mixed with 0.5 mL of 0.1% FeCl_3_ solution to record the absorbance at 700 nm (model Spectro-4; Laby Instruments Industry) against a prepared reagent blank. A standard curve was prepared using l-ascorbic acid and the results were expressed in milligrams of ascorbic acid equivalents (AAE) per gram of dry sample.

#### Ferric reducing antioxidant power

Ferric reducing antioxidant power (FRAP) was determined as per the method given by Tomasina *et al*. (*17*). A volume of 0.1 mL of extract was mixed with 2.4 mL of freshly prepared FRAP reagent followed by the incubation for 5 min at 37 °C. The absorbance was recorded at 620 nm (model Spectro-4; Laby Instruments Industry) against reagent blank. Here, FRAP reagent was prepared by mixing 20 mM FeCl_3_ solution, acetate buffer (300 mM, pH=3.6) and 10 mM TPTZ (2,4,6-Tris(2-pyridyl)-*s*-triazine) solution in the ratio 1:10:1. Trolox was utilized as standard and the results were recorded in micromoles of TE per gram of dry sample.

#### Metal (Fe^2+^) chelating activity analysis

Fe^2+^ chelating activity analysis was performed according to Chew *et al*. (*18*) with certain modifications. The method involved the dilution of 20 µL extract with 5 mL distilled water followed by the addition of 50 µL of FeSO_4_·7H_2_O (0.30 mM). After 7 min, 75 µL of ferrozine solution (0.80 mM) were added and the mixture was allowed to stand for 15 min. Absorbance was recorded at 562 nm (model Spectro-4; Laby Instruments Industry) against aqueous methanol. The following equation was used for the calculation of the percentage of chelating activity:

Chelating activity=(1-*A*_sample_/*A*_control_)·100 /1/

where *A*_sample_ and *A*_control_ are the absorbances recorded for sample and control, respectively. Aqueous methanol was replaced with extract in the control.

#### Antioxidant potency composite index

An equal mass was used for all the antioxidant assays (DPPH, ABTS, FRAP, reducing power and metal chelating activity) and an index value of 100 was assigned to the best score for each test. Thereafter, index scores were calculated for other samples within the test using the following equation:

Antioxidant index score=(sample score/best score)·100 /2/

Antioxidant potency composite (APC) index was calculated by taking the average of all five tests (*19*).

### Phenolic composition

As per the method described by Xiao *et al*. (*10*), samples were extracted with 80% methanol (1:40) at 50 °C for 4 h followed by cooling to room temperature. After centrifuging the extracts at  15 000×*g* (model BS-SP-70BL; Biogenix System, Delhi, DL, India) for 15 min, the solvent was evaporated under reduced pressure and the obtained residue was dissolved in HPLC grade methanol (80%). The final solution was filtered through syringe filter (0.45 µm PVDF membrane) and injected to HPLC (Agilent Technologies, Wilmington, DE, USA) equipped with a reverse-phased ZORBAX Eclipse XDB-phenyl column (4.6 mm×250 mm, 5 µm particle size; Agilent Technologies) with the gradient elution solution A containing 80% acetonitrile, 2% acetic acid, 18% water and solution B containing 8% acetonitrile, 2% acetic acid and 90% water. The other parameters were adjusted as follows: solvent flow rate 1 mL/min, column oven temperature 25 ºC and injection volume 20 µL. Detection of gallic, syringic and cinnamic acids was performed at 276 nm, of chlorogenic, caffeic and ferulic acids at 325 nm, and of *p*-hydroxybenzoic and vanillic acids at 257 nm (model Spectro-4; Laby Instruments Industry). Results were expressed as mass fractions (g per 100 g of sample).

### Determination of antinutrients

Phytic acid was analysed according to the method of Haug and Lantzsch (*20*). Samples (1 g) were extracted with 0.2 M HCl at room temperature. After extraction, stoppered test tubes containing 0.5 mL extract and 1 mL iron(III) solution (0.2 g NH_4_Fe(SO_4_)_2_·12H_2_O in 100 mL of 2 M HCl diluted to 1 L with distilled water) were heated in boiling water bath for 30 min, keeping the tubes well stoppered for the first 5 min. After heating, the tubes were cooled for 15 min in ice water and allowed to adjust to room temperature. Then, 2 mL of 2,2’-bipyridine solution (10 g 2,2’-bipyridine and 10 mL thioglycolic acid dissolved in distilled water and diluted to 1 L) were added followed by thorough mixing. After 30 min, absorbance was recorded at 519 nm (model Spectro-4; Laby Instruments Industry). Sodium phytate was utilized to obtain the standard curve and the results were calculated in milligrams of phytate per gram of dry sample.

Saponins were extracted using the method of Fenwick and Oakenfull (*21*). Defatted samples (1 g) with methanol were kept at room temperature for 24 h. After centrifuging the contents at 1600×*g* (model T-8BL; Laby Instruments Industry), the supernatant was collected and final volume was made to 10 mL with methanol. For determination of saponins, Baccou *et al*. (*22*) method was used wherein 1 mL of extract was kept in a boiling water bath to remove the methanol. After cooling, 2 mL of ethyl acetate were added, followed by the addition of 1 mL of reagent (0.5 mL anisaldehyde mixed with 99.5 mL ethyl acetate) and 1 mL of concentrated sulphuric acid. The contents were thoroughly mixed and allowed to stand for 10 min at room temperature. Absorbance was recorded at 430 nm (model Spectro-4; Laby Instruments Industry) against reagent blank and the results were expressed in milligrams of saponin per gram of dry sample.

Tannins were determined using the modified AOAC method 952.03 (*23*). For extraction, 1 g of sample was refluxed with 40 mL of 10% methanol for 2 h. The contents were centrifuged at 1600×*g* (model T-8BL; Laby Instruments Industry) and the final volume was made to 50 mL with methanol. For determination of tannins, 1 mL of extract was mixed with 7.5 mL of distilled water in test tube followed by addition of 0.5 mL Folin-Denis reagent. Then, 1 mL of saturated Na_2_CO_3_ was added and the contents were thoroughly mixed. After 30 min, absorbance was measured at 760 nm (model Spectro-4; Laby Instruments Industry) against reagent blank. The results were expressed in milligrams of tannic acid equivalents per gram of dry sample.

The isolation and estimation of trypsin inhibitor were performed using the methods of Hajela *et al*. (*24*) and Nath *et al*. (*25*) with slight modifications. A mass of 1 g of the sample was soaked overnight in 10 mL of 0.01 M phosphate buffer (pH=7.5) containing 0.1 M NaCl. The contents were centrifuged at 4850×*g* (model T-8BL; Laby Instruments Industry) for 30 min. The supernatant was heated at 80 °C for 20 min and centrifuged again at 4850×*g* for 30 min. Bovine pancreatic trypsin was used as the enzyme source and the substrate was *N*-benzoyl-dl-arginine-*p*-nitroanilide (BApNA). To 100 µL of the extract, 100 µL of the enzymatic solution were added, followed by the addition of 200 µL of 0.01 M Tris-HCl buffer (pH=7.5) containing 0.02 M CaCl_2_. The content was incubated at 37 °C for 10 min. A volume of 2 mL of the substrate solution (1 mM solution of 9 mg BApNA was prepared by dissolving it first in 2 mL dimethyl sulfoxide (DMSO) and the contents were further diluted to required volume using 0.01 M Tris-HCl buffer, pH=7.5) was added and the content was incubated again at 37 °C for 10 min. The reaction was terminated by the addition of 400 µL of 30% acetic acid. Blank was prepared by adding acetic acid before the substrate. Control was prepared by adding 0.01 M Tris-HCl buffer (pH=7.5) containing 0.02 M CaCl_2_ instead of the extract. Protease inhibition activities were measured by recording the absorbance of the test and control reaction mixtures at 410 nm (model Spectro-4; Laby Instruments Industry). Comparison of the absorbances of test and control mixtures determined the units of inhibited trypsin. Decrease in the absorbance (*A*) measured at 410 nm after 1 min as compared to control of 0.01 *A* was considered to be one unit inhibited per mL of the reaction medium. Results were expressed as trypsin inhibitor activity (TIA) per milligram of dry sample.

### Mineral composition and bioavailability

Each sample was digested with a solution of distilled nitric and perchloric acid (3:1) for 45 min at 90–95 °C. Digested solution was diluted with distilled water, followed by filtration. Final solution was analyzed using inductively coupled plasma optical emission spectrometry (ICP-OES model iCAP 6300; Thermo Fisher Scientific, Waltham, MA, USA) (*26*).

Phytate/mineral molar ratios were determined before and after fermentation to estimate mineral bioavailability. The molecular mass of phytate of 660 g/mol was used for calculation of Phy:Ca, Phy:Zn and Phy:Fe molar ratios (*27*).

### FTIR analysis

FTIR spectrum of samples was obtained using Nicolet 6700 FTIR spectrometer (Thermo Fisher Scientific), operating in the middle IR region (*ṽ*=500–4000 cm^−1^), with attenuated total refection (ATR) mode.

### Statistical analysis

All the analyses were carried out in triplicates and the results were expressed as mean value±standard deviation. To compare the changes before and after fermentation, data sets were subjected to paired *t* test at p<0.05 significance level using SPSS v. 18.0 (*28*). Pearson’s correlation coefficient was performed for correlation analysis.

## RESULTS AND DISCUSSION

### Effect of fermentation on the antioxidant properties of legumes

#### Total phenolic and flavonoid content of legumes

Phenolics are ordinally correlated with the antioxidant properties due to their electron donating ability. Table 1 shows the total phenolic and total flavonoid contents (TPC and TFC, respectively) of unfermented and fermented legumes. Fermentation significantly (p<0.05) enhanced the TPC of all the legumes. Fermented soybean had the highest TPC, 482% higher than unfermented soybean. Fermented kabuli and desi chickpeas were found to contain 52 and 23% higher TPC than the unfermented kabuli and desi chickpeas, respectively. The lowest but significant (p<0.05) improvement was observed in pigeon pea, where the fermented sample contained 12% higher TPC than the unfermented one. The results are consistent with the findings of Xiao *et al*. (*10*) and Lee *et al*. (*29*), who reported 80 and 39% higher TPC in the chickpea fermented by *Cordyceps militaris* SN-18 and pigeon pea fermented by *Bacillus subtilis* BCRC 14715, respectively. Phenolics exist as soluble free esters, and to a major extent, as insoluble esters complexed with plant cell walls, proteins and polysaccharides (*30*). As explained by Juan and Chou (*31*) and Vong *et al*. (*32*), the enzymes β-glucosidases and β-glucuronidases produced by *R. oligosporus* during fermentation of legumes could hydrolyze these complexed phenolics to soluble forms, which is reflected as enhanced phenolic content.

**Table 1:** Total phenolic content expressed as GAE and flavonoid content expressed as QE of unfermented and fermented legumes

*w*/(mg/g)	Kabuli chickpea			Desi chickpea			Pigeon pea			Soybean	
Unfermented	Fermented		Unfermented	Fermented		Unfermented	Fermented		Unfermented	Fermented
GAE	(1.05±0.02)^b^	(1.6±0.1)^a^		(1.01±0.03)^b^	(1.24±0.03)^a^		(3.24±0.04)^b^	(3.64±0.04)^a^		(0.87±0.06)^b^	(5.07±0.06)^a^
QE	(13.0±0.9)^b^	(24.2±0.5)^a^		(16.9±0.9)^b^	(27.1±0.9)^a^		(33.1±0.9)^a^	(32.0±0.4)^a^		(11.4±0.8)^b^	(21.7±0.8)^a^

Results are expressed as mean value±standard deviation of three replicates. Different letters in superscripts represent significant difference (p<0.05) between fermented and unfermented legumes. GAE=gallic acid equivalent, QE=quercetin equivalent

Flavonoids are also correlated with antioxidant properties and their consumption reduces the risk of various diseases, like heart disease and cancer. As shown in Table 1, TFC of kabuli and desi chickpeas, and soybean significantly (p<0.05) increased by 86, 60 and 90% respectively, after the fermentation, while insignificant difference was found between the TFC of fermented and unfermented pigeon peas. In similar context, Juan and Chou (*31*) and Lee *et al*. (*29*) reported 31 and 120% higher TFC in soybean and pigeon pea fermented with *Bacillus subtilis* BCRC 14715 and attributed such enhancement to the activity of microbial enzymes which released bound flavonoids from the substrate. Adetuyi and Ibrahim (*33*) have reported fermentation as effective approach in enhancing the flavonoid content of okra seeds. It was suggested that during fermentation, the rise in matrix acidity led to liberation of bound flavonoids to available forms. However, the studies conducted on cowpea seeds and pistachio hulls gave contradictory results and it have been explained that with the passage of fermentation time, the microorganisms start utilizing available flavonoids, which reduces their concentration (*4*, *34*). Thus, in the present study, the insignificant decrease in the TFC of pigeon pea might be due to the utilization of flavonoids by microorganism for their growth.

#### Antiradical capacity of samples

**Fig. 1:**
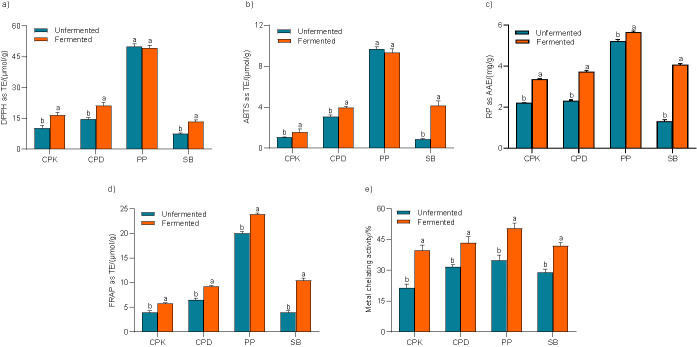
Antioxidant capacity of samples measured as: a) DPPH radical scavenging activity, b) ABTS radical cation scavenging activity, c) reducing power (RP), d) ferric reducing antioxidant power (FRAP) and e) metal chelating activity. Error bars represent the standard deviation of mean values of three replicates. Different letters on bars indicate significant differences (p<0.05) between unfermented and fermented legume. CPK=kabuli chickpea, CPD=desi chickpea, PP=pigeon pea, SB=soybean, TE=Trolox equivalent, AAE=ascorbic acid equivalent

DPPH^•^ and ABTS^+•^ are free radicals which are reduced and stabilized in the presence of electron-donating compounds. Such phenomenon was used for the measurement of antiradical capacity of the samples. Results obtained for DPPH^•^ and ABTS^+•^ scavenging activities are in Figs. 1a and 1b, respectively. A significant difference (p<0.05) was observed between the scavenging activities of fermented and unfermented samples of kabuli and desi chickpeas, and soybean. In terms of DPPH^•^ and ABTS^+•^ inhibition, the highest increment was recorded in soybean, *i.e.* 76 and 370%, respectively, followed by kabuli (61 and 45%) and desi chickpeas (46 and 29%). Pigeon pea was found to have excellent radical scavenging activity; however, the effect of fermentation on such properties was insignificant.

The ability of fermentation to enhance the radical scavenging activity has been reported by various investigators. For instance, Lee *et al*. (*29*) and Juan and Chou (*31*) observed higher scavenging of radicals in fermented pigeon pea and soybean. Similarly, Xiao *et al*. (*10*) reported 48 and 38% higher DPPH^•^ and ABTS^+•^ scavenging activities in chickpea fermented by *Cordyceps militaris* SN-18. In another study, Xiao *et al*. (*35*) observed 44 and 362% higher DPPH^•^ and ABTS^+•^ scavenging activities in mung beans fermented by *Cordyceps militaris* SN-18. These investigations have attributed better scavenging abilities in fermented legumes to the enrichment in phenolic and flavonoid content during fermentation. Thus, the enhanced TPC and TFC in fermented kabuli and desi chickpeas and fermented soybean might be the reason behind their better radical scavenging activities. Similar to the insignificant change in the TFC of pigeon pea, fermentation also had insignificant impact on its radical scavenging activities. In addition, Xiao *et al*. (*10*) demonstrated that the significant enhancement in the inhibition of DPPH and ABTS radicals in fermented chickpeas was ascribed to the enzymatic conversion of phenolic glycosides to more antiradical aglycones. This might also be the reason behind the enhanced radical scavenging activities in the fermented kabuli and desi chickpeas, and especially fermented soybean, which is an excellent source of these compounds.

#### Reducing power of legumes

Reducing power of unfermented and fermented legumes is shown in Fig. 1c. Fermentation significantly (p<0.05) enhanced the reducing ability of all the legumes, with soybean showing the highest increment of 210%, followed by kabuli (51%) and desi (61%) chickpeas and pigeon pea (9%). In similar studies, Xiao *et al*. (*10*) and Xiao *et al*. (*30*) reported 2- to 3-fold higher reducing power in chickpeas and oats fermented by *Cordyceps militaris* SN-18 and correlated it with the reductone-enhancing ability of fermentation. Xiao *et al*. (*30*) explained that these reductones have the ability to stabilize free radicals and thus obstruct toxic radical reactions. In addition, Xiao *et al*. (*35*) ascribed strong reducing ability of fermented mung beans to enhanced phenolics. Thus, it seems that the enhanced phenolic and flavonoid contents in fermented kabuli and desi chickpeas, pigeon pea, and soybean are responsible for their strong reducing power.

#### Ferric reducing antioxidant power of legumes

FRAP reflected the antioxidant potential of food matrices owing to their ability to reduce the complex from TPTZ-Fe^3+^ to TPTZ-Fe^2+^. Fig. 1d shows that the fermentation significantly (p<0.05) increased the FRAP of all the legumes. The highest increase was in soybean (163%), followed by kabuli (46%) and desi (43%) chickpeas, and pigeon pea (19%). This is consistent with the findings of Razak *et al*. (*36*), who reported higher FRAP values in *R. oligosporus-* and *Monascus purpureus-*fermented cereals and attributed it to enhanced phenolic contents during fermentation. Similarly, Xiao *et al*. (*35*) demonstrated a strong correlation between the FRAP and phenolic content of fermented mung beans. They explained that the phenolic compounds enriched during fermentation were responsible for higher reduction of TPTZ-Fe^3+^ to TPTZ-Fe^2+^, resulting in enhanced FRAP. Conclusively, the increment in the FRAP of kabuli and desi chickpeas, pigeon pea and soybean after fermentation with *R. oligosporus* was ascribed to the enrichment of the phenolics as observed in the present study.

#### Metal chelating activity of samples

Fe^2+^ and Cu^2+^ are the major metal ions that initiate the radical chain reactions and catalyze the generation of toxic radicals. Chelating agents can complex the transition metal ions to stabilize them and prevent the body from oxidative damage (*37*). As demonstrated in Fig. 1e, fermentation significantly (p<0.05) improved the metal chelating activity of all legumes, with the highest increase of 85% in kabuli chickpea followed by pigeon pea (45%), soybean (44%) and desi chickpea (37%). The results are in agreement with the findings of Li *et al*. (*38*), who reported 329% higher metal chelating activity in whole soybean flour fermented with *Lactobacillus casei*. It was suggested that the increase in the phenolic content during fermentation was responsible for strong chelating ability. Similarly, Xiao *et al*. (*35*) demonstrated the positive impact of fermentation on the chelating activity of legumes. However, the inconsistency between the chelating ability and corresponding phenolic content has been reported. Similar was observed in the present study; for instance, pigeon pea showed the second highest enhanced metal chelating activity after fermentation, while the lowest improved TPC was recorded in the same extract. It seems that certain other compounds (such as tocopherols or ascorbic acid) might contribute to the metal chelation (*35*).

#### Antioxidant potency composite index of unfermented and fermented legumes

Since five methods with varying principles were employed for the determination of antioxidant activities, the estimation of antioxidant potency composite (APC) index (Table 2) from the indices of the different assays is necessary. The antioxidant potency of all the legumes enhanced with fermentation. Fermented pigeon pea was found to possess the highest antioxidant potency (99) and even unfermented pigeon pea exhibited superior potency (89), thereby showing excellent antioxidant potential of this legume. In fermented kabuli and desi chickpeas, and fermented soybean, the antioxidant potency is in the range of 42–54, which is approx. 2-fold higher than in the corresponding unfermented legumes.

**Table 2:** Antioxidant potency composite index of unfermented and fermented legumes

Index	Kabuli chickpea			Desi chickpea			Pigeon pea			Soybean	
Unfermented	Fermented		Unfermented	Fermented		Unfermented	Fermented		Unfermented	Fermented
DPPH	20.68	33.30		29.18	42.55		100	98.55		15.16	26.74
ABTS	11.04	15.99		31.64	40.90		100	96.57		9.18	43.18
RP	39.13	59.22		40.95	65.85		91.93	100		23.15	71.73
FRAP	16.54	24.09		26.98	38.55		84.07	100		16.70	43.91
MCA	42.55	78.72		62.77	86.17		69.15	100		57.45	82.98
APC	25.99	42.26		38.30	54.81		89.03	99.02		24.33	53.71

DPPH=2,2-diphenyl-1-picrylhydrazyl, ABTS=2,2′-azino-bis(3-ethylbenzothiazoline-6-sulfonic acid), RP=reducing power, FRAP=ferric reducing antioxidant power, MCA=metal chelating activity, APC=antioxidant potency composite index

### Effect of fermentation on the phenolic composition

Eight phenolic compounds were quantified using HPLC to elucidate the effect of fermentation (Table 3). The mass fractions of *p*-hydroxybenzoic acid and chlorogenic acid in kabuli and desi chickpeas, and soybean increased with fermentation, while in pigeon pea, *p*-hydroxybenzoic acid increased but chlorogenic acid was not detected even after its fermentation. Also, higher mass fractions of caffeic acid in fermented kabuli chickpea and pigeon pea, ferulic acid in fermented kabuli chickpea and soybean, gallic acid in fermented desi chickpea and pigeon pea, syringic acid in fermented desi chickpea, pigeon pea and soybean, and vanillic acid in fermented kabuli and desi chickpeas were observed. However, mass fractions of a few compounds were reduced with fermentation, for instance, cinnamic and syringic acids in kabuli chickpea, caffeic, cinnamic and ferulic acids in desi chickpea, cinnamic and ferulic acids in pigeon pea, while reducing trend of any compound was not observed in soybean. Xiao *et al*. (*10*) and Saharan *et al*. (*39*) have demonstrated the ability of fermentation to enhance the mass fractions of phenolic compounds and corresponding antioxidant properties. Xiao *et al*. (*30*) reported higher contents of gallic, ferulic, chlorogenic, caffeic and *p*-hydroxybenzoic acid and vanillin in fermented oats. This was attributed to the activity of microbial enzymes that liberated the complexed phenolic compounds. Furthermore, the strong antioxidant activity in fermented oats was correlated with enhanced mass fractions of these phenolic compounds. Xiao *et al*. (*10*) discussed that the higher content of chlorogenic acid in fermented chickpeas was the major contributor to the strong antioxidant activity owing to its antioxidant potency. Conclusively, higher antioxidant activity in fermented kabuli and desi chickpeas, pigeon pea and soybean could be attributed to the enhanced mass fractions of specific phenolic compounds, especially chlorogenic acid.

**Table 3:** Phenolic composition of unfermented and fermented legumes

Acid	Kabuli chickpea			Desi chickpea			Pigeon pea			Soybean	
Unfermented	Fermented		Unfermented	Fermented		Unfermented	Fermented		Unfermented	Fermented
*w*/(mg/100g)											
Caffeic	N.D.	4.0		0.7	N.D.		0.3	0.4		N.D.	N.D.
Cinnamic	10.3	4.5		27.5	N.D.		3.6	N.D.		N.D.	N.D.
Chlorogenic	N.D.	1.0		0.8	56.0		N.D.	N.D.		N.D.	109.9
Ferulic	18.8	21.1		5.7	5.4		2.2	1.1		0.8	11.3
Gallic	N.D.	N.D.		N.D.	20.6		4.7	33.7		N.D.	N.D.
Syringic	1.4	N.D.		5.3	9.5		12.8	30.1		10.1	86.7
Vanillic	N.D.	1.4		35.6	88.8		N.D.	N.D.		N.D.	N.D.
*p*-Hydroxybenzoic	2.6	158.7		2.6	61.5		1.3	28.8		26.6	46.2

N.D.=not detected

### Effect of fermentation on the antinutrients

**Fig. 2:**
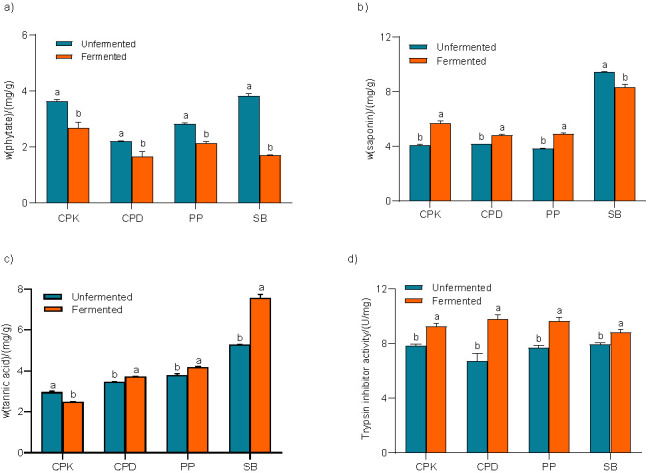
Mass fractions of: a) phytic acid, b) saponins, c) tannins, and d) trypsin inhibitor activity. Error bars represent the standard deviation of mean values of three replicates. Different letters on bars indicate significant difference (p<0.05) between unfermented and fermented legume. CPK=kabuli chickpea, CPD=desi chickpea, PP=pigeon pea, SB=soybean

Phytic acid mass fraction of all legumes significantly (p<0.05) decreased after fermentation (Fig. 2a). The highest reduction of 55% was observed in soybean, followed by kabuli chickpea (27%), pigeon pea (24%) and desi chickpea (24%). Vong *et al*. (*32*) have demonstrated the ability of *R. oligosporus* fermentation to reduce the phytic acid in okara. Similarly, Ranjan *et al*. (*40*) reported 3.2% lower phytate content in rice bran after fermentation with *R. oryzae*. These investigations stated that during fermentation in moderate acidic environment, the activity of endogenous phytase increased, which hydrolyzes the phytic acid present in the substrate. Thus, the lower phytic acid mass fraction measured in fermented kabuli and desi chickpeas, pigeon pea, and soybean could be due to the enhanced activity of phytases during fermentation. Since phytic acid can chelate divalent ions such as Fe^2+^, Mg^2+^, Zn^2+^ and Ca^2+^ and make them unavailable (*40*), fermentation could be an effective approach to degrade phytic acid and liberate bound nutrients.

Saponins in fermented kabuli and desi chickpeas, and fermented pigeon pea were found to be 40, 16 and 28%, respectively, higher (p<0.05) than in their unfermented counterparts (Fig. 2b). Xiao *et al*. (*10*) reported a similar result that saponins in chickpeas increased 3-fold after fermentation with *Cordyceps militaris* SN-18 and attributed it to the synthesis of saponins as defence mechanism during fermentation. Conversely, the saponin content of soybean significantly (p<0.05) decreased (12%) with fermentation. This is in agreement with the findings of Rui *et al.* (*41*), who stated that reduced saponin content in soybean fermented with *Lactobacillus* plantarum B1-6 was due to β-glucosidase produced during fermentation, which hydrolyzed the saponin molecules to sapogenins and sugar side chains and reduced their solubility. Saponins are usually reported as antinutrients due to their haemolytic and membranolytic activities (*41*). However, they are also considered as bioactive components owing to their antioxidant properties (*10*).

Fermentation caused 8, 10 and 43% increase (p<0.05) in the tannins of desi chickpea, pigeon pea and soybean respectively, while in kabuli chickpea, the values were reduced (p<0.05) by 16% (Fig. 2c). In a similar investigation, Osman (*42*) observed 350% higher tannin content in fermented pearl millet. This was attributed to the enzymatic hydrolysis of condensed tannins. Conversely, Egounlety and Aworh (*43*) and James *et al*. (*44*) have reported reducing effect of fermentation on the tannin content of legumes. This could be due to the degradation of tannins by polyphenol oxidase and/or tannase enzymes produced during fermentation (*44*).

Trypsin inhibitor activity in kabuli and desi chickpeas, pigeon pea and soybean increased by 18, 46, 25 and 11% respectively, after fermentation with *R. oligosporus* (Fig. 2d). This is in agreement with the findings of Egounlety and Aworh (*43*), who reported increased trypsin inhibitor activity in *R. oligosporus*-fermented soybean. Prinyawiwatkul *et al*. (*45*) stated that 35% higher trypsin inhibitory activity in *R. microsporus*-fermented cowpea was due to fungal protease that released the active trypsin inhibitors from heat-resistant, inactive bound form. During fermentation, the activity of fungal protease exposes the amino acid residues resulting in the solubilization of heat-denatured trypsin inhibitors that form a complex with trypsin. However, complete inactivation of these released trypsin inhibitors can be achieved by adequately cooking the flour before consumption (*45*). Contrarily, some investigations have reported reduced level of trypsin inhibitors after fermentation, attributing it to the activity of hydrolytic enzymes produced during fermentation (*40*, *41*).

### Correlation analysis

Pearson’s correlation coefficients among bioactive constituents, antioxidant activities and antinutrients of unfermented and fermented legumes were determined (Table 4). A strong positive correlation (p<0.05) was found between TFC and different antioxidant activities such as DPPH^•^ (r=0.884), ABTS^+•^ (r=0.867), reducing power (r=0.950), FRAP (r=0.860) and metal chelating activity (r=0.761). Compared to TFC, TPC was moderately correlated with these antioxidant activities. The positive correlation among TPC, TFC and antioxidant activities demonstrated the contribution of phenolics to antioxidant potential of legumes. Besides, DPPH^•^ and ABTS^+•^ demonstrated a strong correlation (r=0.963, p<0.01). Except metal chelation, other antioxidant activities had strong correlation with each other. Antinutrients (phytic acid, saponins and tannins) are also considered as bioactive constituents; however, they were found to be poorly correlated with antioxidant activities in the present study.

**Table 4:** Correlation among bioactive constituents, antioxidant activities and antinutrients of unfermented and fermented legumes

	TPC	TFC	DPPH	ABTS	RP	FRAP	MCA	Phytic acid	Saponins	Tannins
TPC	1.000	0.547	0.476	0.631	0.738*	0.657	0.578	-0.498	0.175	0.657
TFC		1.000	0.884**	0.867**	0.950**	0.860**	0.761*	-0.564	-0.425	-0.096
DPPH			1.000	0.963**	0.883**	0.960**	0.536	-0.251	-0.477	-0.145
ABTS				1.000	0.908**	0.981**	0.578	-0.392	-0.383	0.084
RP					1.000	0.923**	0.756*	-0.563	-0.359	0.080
FRAP						1.000	0.648	-0.374	-0.315	0.099
MCA							1.000	-0.774*	0.026	0.228
Phytic acid								1.000	0.157	-0.268
Saponins									1.000	0.735*
Tannins										1.000

TPC=total phenolic content, TFC=total flavonoid content, DPPH= 2,2-diphenyl-1-picrylhydrazyl, ABTS=2,2′-azino-bis(3-ethylbenzothiazoline-6-sulfonic acid), RP=reducing power, FRAP=ferric reducing antioxidant power, MCA=metal chelating activity. Significant at *p<0.05, **p<0.01

### Effect of fermentation on the mineral composition and bioavailability

Nine minerals (P, Mg, Na, K, Cu, Mn, Ca, Fe and Zn) were quantified using ICP-OES. Fermentation significantly (p<0.05) enhanced the mass fraction of minerals in all the legumes (Table 5), which has also been observed by other investigators. For instance, Ali *et al*. (*46*) reported the higher contents of macro- and microminerals in soybean fermented with *Bacillus subtilis*. Asres *et al.* (*27*) discussed that higher mineral contents in fermented legumes were ascribed to the activity of microbial enzymes that released the minerals from chelated complex compounds. Dhull *et al.* (*47*) discussed that phytates are mainly responsible for impaired bioavailability of minerals and attributed enhanced content of minerals (Ca, Na, K, Cu, Fe and Zn) in *Aspergillus awamori*-fermented lentil to degradation of phytates during fermentation.

**Table 5:** Mineral composition and their bioavailability in unfermented and fermented legumes

Mineral	Kabuli chickpea			Desi chickpea			Pigeon pea			Soybean	
Unfermented	Fermented	Unfermented	Fermented	Unfermented	Fermented	Unfermented	Fermented
*w*/(mg/100 g)										
Phosphorous	(111.3±8.0)^b^	(278.5±9.2)^a^		(83.0±7.7)^a^	(86.4±6.3)^a^		(74.8±5.5)^b^	(115.9±8.4)^a^		(196.2±9.9)^b^	(299.13±10.08)^a^
Magnesium	(98.9±4.4)^b^	(126.4±5.4)^a^		(67.0±5.6)^a^	(68.5±4.8)^a^		(45.4±3.9)^b^	(93.8±7.7)^a^		(76.3±5.5)^b^	(120.4±7.7)^a^
Sodium	(18.1±1.7)^b^	(26.0±2.4)^a^		(20.9±2.1)^b^	(26.6±.2.63^a^		(7.6±0.7)^b^	(16.6±1.5)^a^		(15.2±1.4)^b^	(26.4±2.1)^a^
Potassium	(431.7±21.7)^b^	(662.0±29.0)^a^		(214.8±16.5)^a^	(213.7±.15.8)^a^		(757.4±20.1)^b^	(981.0±26.4)^a^		(335.2±11.7)^b^	(589.6±15.2)^a^
Copper	(0.78±0.01)^b^	(1.53±0.08)^a^		(0.68±0.05)^b^	(0.93±0.09)^a^		(2.2±0.2)^b^	(4.7±0.5)^a^		(0.88±0.09)^b^	(1.9±0.11^a^
Manganese	(8.3±0.8)^b^	(20.7±2.1)^a^		(5.8±0.4)^a^	(6.8±0.5)^a^		(4. 7±0.5)^b^	(7.1±0.7)^a^		(1.5±0.1)^b^	(3.1±0.5)^a^
Calcium	(40.4±3.0)^b^	(81.55±4.07)^a^		(21.2±2.0)^a^	(21.3±2.2)^a^		(17.2±1.9)^b^	(44.4±3.7)^a^		(75.0±5.6)^b^	(117.6±7.7)^a^
Iron	(5.1±0.4)^b^	(6.3±0.8)^a^		(4.7±0.3)^b^	(5.9±0.6)^a^		(11.9±1.1)^b^	(21.2±2.0)^a^		(16.3±1.0)^b^	(31.1±2.2)^a^
Zinc	(2.9±0.2)^b^	(4.2±0.4)^a^		(5.2±0.5)^a^	(5.2±0.4)^a^		(3.2±0.3)^b^	(6.9±0.6)^a^		(2.8±0.3)^b^	(4.4±0.6)^a^
*r*_phytate,metal_											
Calcium	0.55	0.20		0.63	0.47		1.01	0.29		0.31	0.09
Iron	6.09	3.59		3.99	2.40		2.01	0.86		1.98	0.46
Zinc	12.32	6.31		4.19	3.19		8.69	3.08		13.76	3.80

Results are expressed as mean value±standard deviation of three replicates. Different letters in superscripts show significant difference (p<0.05) between unfermented and fermented legume

Furthermore, the bioavailability of specific minerals, *i.e.* Ca, Fe and Zn, was assessed by calculating the phytate/mineral molar ratios (Table 5). In fermented kabuli chickpea and soybean, the molar ratio of phytate to calcium was below the critical value (0.24), showing good bioavailability of calcium. Similarly, the phyate/iron molar ratio was <1 in fermented pigeon pea and soybean, which indicates good bioavailability of iron. However, the phytate/calcium molar ratio in fermented desi chickpea and pigeon pea and phytate/iron molar ratio in fermented kabuli and desi chickpeas were above critical values, which is indicative of impaired mineral bioavailability. All the fermented and unfermented legumes had superior bioavailability of zinc as their phytate/zinc molar ratio was <15. Overall, the molar ratios of phytate to minerals decreased with fermentation, which signifies the improved bioavailability of minerals in fermented legumes. This is in agreement with the findings of Asres *et al.* (*27*), who reported the enhanced mineral bioavailability in fermented teff and wheat, and attributed it to reduced levels of phytic acid after fermentation.

### FTIR spectra of unfermented and fermented legumes

**Fig. 3:**
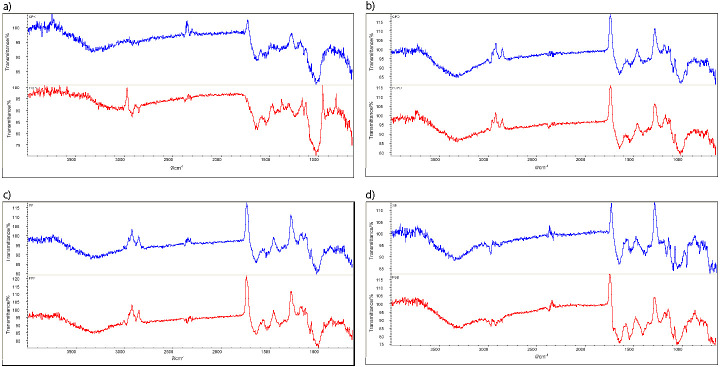
FTIR analysis of unfermented (blue line) and fermented (red line) legumes: a) kabuli chickpea, b) desi chickpea, c) pigeon pea and d) soybean

FTIR analysis was performed to identify the changes induced by fermentation in the structure of legumes using the vibrational pattern of various functional groups present. As shown in figure Fig. 3, identical compounds were present in unfermented and fermented legumes, which is observable in similar patterns of absorption bands. After fermentation, the band shifted to lower wavelengths for all the legumes along with reduction in the intensities. The amide I band (1600–1700 cm^-1^) provides information on the protein secondary structures where β-sheet structure, random coil, α-helix structure and β-turn structures are observed in the ranges 1615–1638, 1638–1645, 1645–1662 and 1662–1682 cm^−1^ respectively (*48*). The changes in the carbohydrate zone (750–1200 cm^-1^ fingerprint region) and amide I zone after fermentation can be attributed to partial protein and starch depolymerization. An intense band around 3300 cm^-1^ was observed for both fermented and unfermented legumes, which may be due to the stretching vibration of O-H, mainly in phenolic groups (*49*). Aromatic groups are inferred by the band around 1407 cm^-1^ (C-C stretch) and the peak near 798 cm^-1^. Slight modification was recorded near these bands in fermented legumes since fermentation was responsible for changes in these functional groups, especially aromatic and phenolic groups, which include antinutritional factors, such as phytic acid, saponins and tannins.

## CONCLUSIONS

Fermentation of some commonly consumed legumes: kabuli and desi chickpeas, pigeon pea and soybean with *Rhizopus* *oligosporus* showed significant changes in the nutritional and antinutritional composition. The total phenolic and flavonoid contents and antioxidant activities increased, and the phenolic composition was improved after fermentation. A strong positive correlation was observed between the flavonoid content and different antioxidant properties. Furthermore, the phytic acid content of legumes was reduced with fermentation, which consequently enhanced the content and bioavailability of the minerals. However, the mass fractions of tannins and saponins and the activity of trypsin inhibitors increased in most of the legumes. FTIR analysis showed the changes in the functional groups of the legumes after fermentation. Based on this study, we conclude that legume fermentation led to superior phenolic composition, enhanced antioxidant properties and higher mineral bioavailability. Thus, fermented legumes can be incorporated as functional ingredients in the development of healthy diets possessing the ability to counter the lifestyle-related oxidative stress, chronic disorders and micronutrient (mineral) deficiency.

## References

[1] Jeong D Han JA Liu Q Chung HJ. Effect of processing, storage, and modification on *in vitro* starch digestion characteristics of food legumes: A review. Food Hydrocoll. 2019;90:367–76. 10.1016/j.foodhyd.2018.12.039

[2] Zafar TA Aldughpassi A Al-Mussallam A Al-Othman A. Microstructure of whole wheat *versus* white flour and wheat-chickpea flour blends and dough: Impact on the glycemic response of pan bread. Int J Food Sci. 2020;2020:8834960. 10.1155/2020/8834960 33083447 PMC7557900

[3] De Pasquale I Pontonio E Gobbetti M Rizzello CG. Nutritional and functional effects of the lactic acid bacteria fermentation on gelatinized legume flours. Int J Food Microbiol. 2020;316:108426. 10.1016/j.ijfoodmicro.2019.108426 31722270

[4] Yadav N Kaur D Malaviya R Singh M Fatima M Singh L. Effect of thermal and non-thermal processing on antioxidant potential of cowpea seeds. Int J Food Prop. 2018;21(1):437–51. 10.1080/10942912.2018.1431659

[5] Maleki S Razavi SH. Pulses’ germination and fermentation: Two bioprocessing against hypertension by releasing ACE inhibitory peptides. Crit Rev Food Sci Nutr. 2021;61(17):2876–93. 10.1080/10408398.2020.1789551 32662284

[6] Chinma CE Azeez SO Sulayman HT Alhassan K Alozie SN Gbadamosi HD Evaluation of fermented African yam bean flour composition and influence of substitution levels on properties of wheat bread. J Food Sci. 2020;85(12):4281–9. 10.1111/1750-3841.15527 33216358

[7] Cantabrana I Perise R Hernández I. Uses of *Rhizopus oryzae* in the kitchen. Int J Gastron Food Sci. 2015;2(2):103–11. 10.1016/j.ijgfs.2015.01.001

[8] Ibarruri J Cebrián M Hernández I. Solid state fermentation of brewer’s spent grain using *Rhizopus* sp. to enhance nutritional value. Waste Biomass Valoriz. 2019;10(12):3687–700. 10.1007/s12649-019-00654-5

[9] Collados A Conversa V Fombellida M Rozas S Kim JH Arboleya JC Applying food enzymes in the kitchen. Int J Gastron Food Sci. 2020;21:100212. 10.1016/j.ijgfs.2020.100212

[10] Xiao Y Xing G Rui X Li W Chen X Jiang M Enhancement of the antioxidant capacity of chickpeas by solid state fermentation with *Cordyceps militaris* SN-18. J Funct Foods. 2014;10:210–22. 10.1016/j.jff.2014.06.008

[11] Reyes‐Moreno C Cuevas‐Rodríguez EO Milán‐Carrillo J Cárdenas‐Valenzuela OG Barrón‐Hoyos J. Solid state fermentation process for producing chickpea (*Cicer arietinum* L) tempeh flour. Physicochemical and nutritional characteristics of the product. J Sci Food Agric. 2004;84(3):271–8. 10.1002/jsfa.1637

[12] Singleton VL Orthofer R Lamuela-Raventós RM. Analysis of total phenols and other oxidation substrates and antioxidants by means of Folin-Ciocalteu reagent. Methods Enzymol. 1999;299:152–78. 10.1016/S0076-6879(99)99017-1

[13] Dini I Tenore GC Dini A. Antioxidant compound contents and antioxidant activity before and after cooking in sweet and bitter *Chenopodium quinoa* seeds. Lebensm Wiss Technol. 2010;43(3):447–51. 10.1016/j.lwt.2009.09.010

[14] Brand-Williams W Cuvelier ME Berset C. Use of a free radical method to evaluate antioxidant activity. Lebensm Wiss Technol. 1995;28(1):25–30. 10.1016/S0023-6438(95)80008-5

[15] Yilmaz-Ersan L Ozcan T Akpinar-Bayizit A Sahin S. Comparison of antioxidant capacity of cow and ewe milk kefirs. J Dairy Sci. 2018;101(5):3788–98. 10.3168/jds.2017-13871 29477522

[16] Oyaizu M. Studies on products of browning reactions, Antioxidant activities of products of browning reaction prepared from glucose amine. Jap J Nutr. 1986;44(6):307-15 (in Japanese). 10.5264/eiyogakuzashi.44.307 10.5264/eiyogakuzashi.44.307

[17] Tomasina F Carabio C Celano L Thomson L. Analysis of two methods to evaluate antioxidants. Biochem Mol Biol Educ. 2012;40(4):266–70. 10.1002/bmb.20617 22807430

[18] Chew YL Goh JK Lim YY. Assessment of *in vitro* antioxidant capacity and polyphenolic composition of selected medicinal herbs from *Leguminosae* family in Peninsular Malaysia. Food Chem. 2009;116(1):13–8. 10.1016/j.foodchem.2009.01.091

[19] Todorovic V Redovnikovic IR Todorovic Z Jankovic G Dodevska M Sobajic S. Polyphenols, methylxanthines, and antioxidant capacity of chocolates produced in Serbia. J Food Compos Anal. 2015;41:137–43. 10.1016/j.jfca.2015.01.018

[20] Haug W Lantzsch HJ. Sensitive method for the rapid determination of phytate in cereals and cereal products. J Sci Food Agric. 1983;34(12):1423–6. 10.1002/jsfa.2740341217

[21] Fenwick DE Oakenfull D. Saponin content of food plants and some prepared foods. J Sci Food Agric. 1983;34(2):186–91. 10.1002/jsfa.2740340212 6855202

[22] Baccou JC Lambert F Sauvaire Y. Spectrophotometric method for the determination of total steroidal sapogenin. Analyst. 1977;102(1215):458–65. 10.1039/an9770200458 883665

[23] Official Method AOAC. 952.03. Tannin in distilled liquors. Rockville, MD, USA: AOAC International; 2005.

[24] Hajela N Pande AH Sharma S Rao DN Hajela K. Studies on a doubleheaded protease inhibitor from *Phaseolus mungo.* J Plant Biochem Biotechnol. 1999;8(1):57–60. 10.1007/BF03263059

[25] Nath AK Chadha K Sharma P Deepika R. Purification and characterization of a novel inhibitor from *Poinciana pulcherrima* seeds with activity towards pest digestive enzymes. Funct Plant Sci Biotechnol. 2012;6(1):75–81.

[26] Özcan MM Dursun N Juhaimi FA. Macro- and microelement contents of some legume seeds. Environ Monit Assess. 2013;185(11):9295–8. 10.1007/s10661-013-3252-x 23715731

[27] Asres DT Nana A Nega G. Complementary feeding and effect of spontaneous fermentation on anti-nutritional factors of selected cereal-based complementary foods. BMC Pediatr. 2018;18(1):394. 10.1186/s12887-018-1369-3 30579346 PMC6304228

[28] Sahhaf Ebrahimi F Rad AH Mosen M Abbasalizadeh F Tabrizi A Khalili L. Effect of *L. acidophilus* and *B. lactis* on blood glucose in women with gestational diabetes mellitus: A randomized placebo-controlled trial. Diabetol Metab Syndr. 2019;11(1):75. 10.1186/s13098-019-0471-5 31485272 PMC6714347

[29] Lee BH Lai YS Wu SC. Antioxidation, angiotensin converting enzyme inhibition activity, nattokinase, and antihypertension of *Bacillus subtilis* (natto)-fermented pigeon pea. J Food Drug Anal. 2015;23(4):750–7. 10.1016/j.jfda.2015.06.008 28911492 PMC9345443

[30] Xiao Y Rui X Xing G Wu H Li W Chen X Solid state fermentation with *Cordyceps militaris* SN-18 enhanced antioxidant capacity and DNA damage protective effect of oats (*Avena sativa* L.). J Funct Foods. 2015;16:58–73. 10.1016/j.jff.2015.04.032

[31] Juan MY Chou CC. Enhancement of antioxidant activity, total phenolic and flavonoid content of black soybean by solid state fermentation with *Bacillus subtilis* BCRC 14715. Food Microbiol. 2010;27(5):586–91. 10.1016/j.fm.2009.11.002 20510775

[32] Vong WC Hua XY Liu SQ. Solid-state fermentation with *Rhizopus oligosporus* and *Yarrowia lipolytica* improved nutritional and flavour properties of okara. Lebensm Wiss Technol. 2018;90:316–22. 10.1016/j.lwt.2017.12.050

[33] Adetuyi FO Ibrahim TA. Effect of fermentation time on the phenolic, flavonoid and vitamin C contents and antioxidant activities of okra (*Abelmoschus esculentus*) seeds. Niger Food J. 2014;32(2):128–37. 10.1016/S0189-7241(15)30128-4

[34] Ehsan K Ehsan O Rudi H Jaafar HZ. Solid state fermentation effects on pistachio hulls antioxidant activities. King Khalid Univ Res J. 2010;15:260–6.

[35] Xiao Y Zhang Q Miao J Rui X Li T Dong M. Antioxidant activity and DNA damage protection of mung beans processed by solid state fermentation with *Cordyceps militaris* SN-18. Innov Food Sci Emerg Technol. 2015;31:216–25. 10.1016/j.ifset.2015.06.006

[36] Razak DLA Rashid NYA Jamaluddin A Sharifudin SA Long K. Enhancement of phenolic acid content and antioxidant activity of rice bran fermented with *Rhizopus oligosporus* and *Monascus purpureus.* Biocatal Agric Biotechnol. 2015;4(1):33–8. 10.1016/j.bcab.2014.11.003

[37] Zhang Z Lv G Pan H Fan L Soccol CR Pandey A. Production of powerful antioxidant supplements via solid-state fermentation of wheat (*Triticum aestivum* Linn.) by *Cordyceps militaris.* Food Technol Biotechnol. 2012;50(1):32–9.

[38] Li S Jin Z Hu D Yang W Yan Y Nie X Effect of solid-state fermentation with *Lactobacillus casei* on the nutritional value, isoflavones, phenolic acids and antioxidant activity of whole soybean flour. Lebensm Wiss Technol. 2020;125:109264. 10.1016/j.lwt.2020.109264

[39] Saharan P Sadh PK Duhan S Duhan JS. Bio-enrichment of phenolic, flavonoids content and antioxidant activity of commonly used pulses by solid-state fermentation. J Food Meas Charact. 2020;14(3):1497–510. 10.1007/s11694-020-00399-z

[40] Ranjan A Sahu NP Deo AD Kumar S. Solid state fermentation of de-oiled rice bran: Effect on *in vitro* protein digestibility, fatty acid profile and anti-nutritional factors. Food Res Int. 2019;119:1–5. 10.1016/j.foodres.2019.01.054 30884637

[41] Rui X Wang M Zhang Y Chen X Li L Liu Y Optimization of soy solid‐state fermentation with selected lactic acid bacteria and the effect on the anti‐nutritional components. J Food Process Preserv. 2017;41(6):e13290. 10.1111/jfpp.13290

[42] Osman MA. Effect of traditional fermentation process on the nutrient and antinutrient contents of pearl millet during preparation of Lohoh. J Saudi Soc Agric Sci. 2011;10(1):1–6. 10.1016/j.jssas.2010.06.001

[43] Egounlety M Aworh OC. Effect of soaking, dehulling, cooking and fermentation with *Rhizopus oligosporus* on the oligosaccharides, trypsin inhibitor, phytic acid and tannins of soybean (*Glycine max* Merr.), cowpea (*Vigna unguiculata* L. Walp) and groundbean (*Macrotyloma geocarpa* Harms). J Food Eng. 2003;56(2-3):249–54. 10.1016/S0260-8774(02)00262-5

[44] James S Nwabueze TU Ndife J Onwuka GI Usman MA. Influence of fermentation and germination on some bioactive components of selected lesser legumes indigenous to Nigeria. J Agric Food Res. 2020;2:100086. 10.1016/j.jafr.2020.100086

[45] Prinyawiwatkul W Eitenmiller RR Beuchat LR McWatters KH Phillips RD. Cowpea flour vitamins and trypsin inhibitor affected by treatment and fermentation with *Rhizopus microsporus.* J Food Sci. 1996;61(5):1039–42. 10.1111/j.1365-2621.1996.tb10928.x

[46] Ali MW Shahzad R Bilal S Adhikari B Kim ID Lee JD Comparison of antioxidants potential, metabolites, and nutritional profiles of Korean fermented soybean (*Cheonggukjang*) with *Bacillus subtilis* KCTC 13241. J Food Sci Technol. 2018;55(8):2871–80. 10.1007/s13197-018-3202-2 30065396 PMC6046010

[47] Dhull SB Punia S Kidwai MK Kaur M Chawla P Purewal SS Solid‐state fermentation of lentil (*Lens culinaris* L.) with *Aspergillus awamori*: Effect on phenolic compounds, mineral content, and their bioavailability. Legume Sci. 2020;2(3):e37. 10.1002/leg3.37

[48] Hou F Ding W Qu W Oladejo AO Xiong F Zhang W Alkali solution extraction of rice residue protein isolates: Influence of alkali concentration on protein functional, structural properties and lysinoalanine formation. Food Chem. 2017;218:207–15. 10.1016/j.foodchem.2016.09.064 27719899

[49] Sivam AS Sun-Waterhouse D Perera CO Waterhouse GI. Application of FT-IR and Raman spectroscopy for the study of biopolymers in breads fortified with fibre and polyphenols. Food Res Int. 2013;50(2):574–85. 10.1016/j.foodres.2011.03.039

